# Unlocking the Potential of Cranberry (*Vaccinium macrocarpon* Aiton) Pruning Biomass: Phenolic Composition and Antioxidant Properties of Response Surface Methodology Optimized Extracts

**DOI:** 10.3390/molecules31040698

**Published:** 2026-02-17

**Authors:** Tomasz Piechowiak, Ireneusz Kapusta, Maciej Balawejder, Radosław Józefczyk, Natalia Matłok

**Affiliations:** 1 Department of Chemistry and Food Toxicology, University of Rzeszow, St. Ćwiklińskiej 1a, 35-601 Rzeszow, Poland; tpiechowiak@ur.edu.pl (T.P.); mbalawejder@ur.edu.pl (M.B.); rjozefczyk@ur.edu.pl (R.J.); 2 Department of Food Technology and Human Nutrition, University of Rzeszow, St. Zelwerowicza 4, 35-601 Rzeszow, Poland; ikapusta@ur.edu.pl; 3 Department of Food and Agriculture Production Engineering, University of Rzeszow, St. Zelwerowicza 4, 35-601 Rzeszow, Poland

**Keywords:** *Vaccinium macrocarpon* Aiton, cranberry biomass, extract, response surface methodology (RSM), optimization, phenolic profile, bioactive compounds, antioxidant activity, circular bioeconomy, plant by-products, valorization

## Abstract

The aim of this study was to investigate the chemical composition and biological activity of extracts obtained from vegetative cranberry biomass generated during plantation rejuvenation. This biomass, composed mainly of young shoots removed during routine agricultural maintenance, represents a readily available and currently underutilized by-product of commercial cranberry (*Vaccinium macrocarpon* Aiton) cultivation. Extraction optimization was performed using response surface methodology (RSM), which enabled both the assessment of the effects of process variables and the identification of conditions ensuring maximal extraction efficiency. The optimal parameters were determined to be 40% ethanol, a temperature of 60 °C, and an extraction time of 49.44 min, and these conditions were further validated through an additional triplicate extraction. The resulting extract exhibited a high antioxidant activity (429–490 mg Trolox equivalents per gram) and was rich in phenolic compounds, particularly quercetin glycoside derivatives. The chemical profile and bioactivity of the extract highlight the considerable potential of cranberry pruning biomass as an alternative, sustainable source of high-value phytochemicals. Its valorization may support the development of environmentally friendly extraction technologies and contribute to closing the resource loop within agricultural production systems.

## 1. Introduction

Large-fruited cranberry (*Vaccinium macrocarpon* Ait.) is a valuable horticultural plant, widely cultivated due to its high content of bioactive compounds, such as polyphenols, flavonoids, anthocyanins, and organic acids, which exhibit antioxidant, anti-inflammatory, and antibacterial properties. The consumption and industrial processing of cranberries have steadily increased in recent years, driven by growing consumer interest in natural products that promote health and prevent chronic diseases [1,2,3,4,5,6,7,8,9].

The optimal recovery of polyphenols and other bioactive compounds from plant materials, however, requires the proper selection of extraction conditions, such as solvent concentration, temperature, and extraction time. Due to the complex nature of plant matrices and the nonlinear relationships between process parameters and extraction efficiency, multivariate optimization models are increasingly applied. One of the most effective and widely used methods is Response Surface Methodology (RSM), which enables the simultaneous analysis of multiple factors and the identification of conditions that maximize phenolic recovery and antioxidant activity of the extracts. This method has been successfully applied in numerous studies on the extraction of phytochemicals, justifying its use for cranberry pruning biomass as well [10,11,12,13]. The optimization of extraction processes to maximize the recovery of bioactive compounds from plant by-products has been effectively achieved using Response Surface Methodology (RSM). RSM is a powerful design-of-experiments tool that enables the simultaneous evaluation of multiple process variables and their interactions, providing statistically optimized conditions for solvent type, temperature, extraction time, and solvent-to-solid ratios, among other factors. Numerous recent studies have applied RSM to optimize extraction yields and antioxidant properties from fruit and vegetable by-products, including carotenoids, phenolics, and flavonoids, underscoring its utility in process development for sustainable valorization [14,15,16,17].

Overall, the valorization of cranberry by-products using advanced extraction methods, coupled with statistical optimization techniques like RSM, highlights the potential of such waste materials as high-value bioactive sources, contributing both to waste reduction and to the enhancement of health-related properties in derived products.

Recent research has increasingly focused on the valorization of berry by-products generated during food processing, recognizing them as rich sources of bioactive compounds with potential applications in functional foods, nutraceuticals, and sustainable bioproducts. Cranberry pomace, in particular, is abundantly produced during juice and concentrate manufacture, yet remains underutilized despite its high content of polyphenols, dietary fibers, and other phytochemicals with significant antioxidant, anti-inflammatory, and health-promoting properties. Studies have demonstrated that incorporating cranberry pomace into food matrices significantly increases total phenolic content (TPC) and antioxidant capacity and enhances functional properties such as radical-scavenging activity and enzyme inhibitory effects, aligning with circular economy strategies for waste valorization [3]. Plant-derived biomass, including horticultural and fruit-processing wastes, constitutes an important subject of research due to its potential applications in biorefineries, the production of functional materials, and as a raw material for the recovery of bioactive compounds. In this context, large-fruited cranberry (*Vaccinium macrocarpon* Ait.) is of particular importance as a valuable horticultural crop widely cultivated for its high content of bioactive substances, such as polyphenols, flavonoids, anthocyanins, and organic acids, which exhibit antioxidant, anti-inflammatory, and antibacterial properties. Growing consumer interest in natural health-promoting products has contributed to a steady increase in both cranberry consumption and its industrial processing [18].

Comprehensive literature reviews indicate that the chemical composition of cranberry fruits is well documented, encompassing both bioactive fractions and fiber components of nutritional and pharmacological relevance. At the same time, numerous studies focus on processing by-products, such as pomace generated during juice and concentrate production. Cranberry pomace has been shown to be a rich source of polyphenols, dietary fiber, pectins, cellulose, and hemicelluloses, and its incorporation into food products significantly increases total phenolic content (TPC) and antioxidant activity, aligning with circular economy strategies and waste valorization [19].

Despite the extensive interest in fruit composition and processing residues, the literature still insufficiently addresses differences in the composition of biomass derived from other sources associated with cranberry production, particularly biomass generated during agronomic practices [20].

From an agronomic perspective, cranberry plantations require periodic rejuvenation to maintain optimal yield and high fruit quality. Every few years, growers perform mowing or cutting of the aboveground parts of the plants as part of plantation renewal. This practice generates a significant amount of biomass—leaves and shoots—which is usually treated as waste [5]. However, this material can serve as a valuable and renewable source of bioactive compounds. Studies indicate that cranberry vegetative tissues contain significant amounts of phenolic compounds and triterpenoids with strong antioxidant and cytoprotective potential. The utilization of cranberry biomass produced during plantation rejuvenation therefore provides promising opportunities for the sustainable recovery of bioactive substances. Such an approach aligns with the principles of a circular bioeconomy, contributing to waste reduction and the development of high-value natural ingredients for the pharmaceutical, nutraceutical, and cosmetic industries [6,7,8,9]. The present study aimed to characterize the phenolic composition of large-fruited cranberry biomass produced during plantation rejuvenation and to evaluate its antioxidant properties. Furthermore, the study sought to optimize the extraction process of bioactive compounds using Response Surface Methodology (RSM) to identify conditions that maximize both extraction yield and antioxidant activity of the resulting extracts.

## 2. Results and Discussion

The efficiency of the periodic extraction of phytochemicals from plants depends on many factors, the most important of which are the polarity of the solvent used, the process temperature, and the extraction time. According to current reports, the highest degree of antioxidant extraction from plant matrices is achieved using aqueous ethanol or methanol solutions, as they enable the extraction of both polar and medium-polar antioxidants, particularly flavonoid aglycones. Increasing the extraction temperature improves the diffusion coefficient and facilitates solvent penetration into the plant matrix by reducing its viscosity, loosening cell structures, and increasing the solubility of compounds, thus shortening the overall extraction time [21]. Therefore, to obtain an extract with maximum polyphenol content and antioxidant activity, the process must be optimized.

In this study, extraction optimization was performed using response surface methodology (RSM). This mathematical tool, commonly applied in similar research, allows both the assessment of factor effects on the studied variables and the determination of process conditions under which extraction is most efficient [22]. The input variables selected for the experimental design included ethanol concentration in the range of 40–96%, extraction temperature between 25 and 60 °C, and extraction time between 5 and 60 min. Water as a solvent was deliberately excluded due to its low ability to dissolve certain polyphenols and its high capacity to dissolve proteins, including enzymes that degrade antioxidants and may remain active in freeze-dried material. The maximum extraction temperature of 60 °C was selected due to the high volatility of ethanol and the resulting risk of solvent loss during extraction, as well as the potential thermal degradation of phytonutrients.

**Figure 1:**
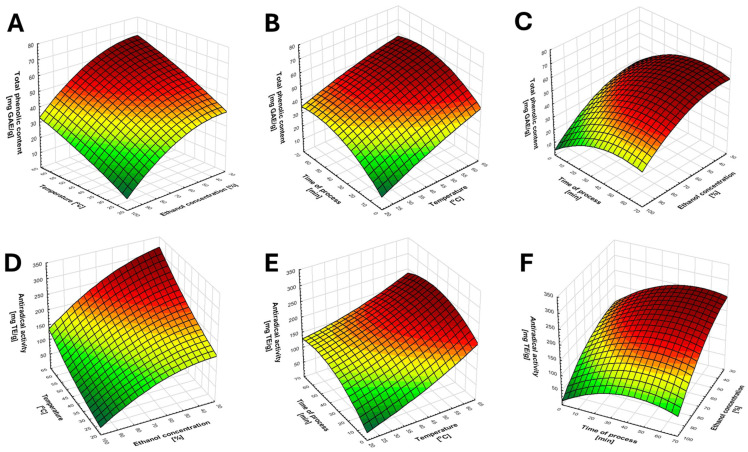
Graphical representation of the relationship between the tested variables. (**A**–**C**) illustrate the effect of the variables on polyphenol recovery, whereas (**D**–**F**) present the effect of the factors on the recovery of antioxidants reacting with DPPH.

As shown in Table 1, within the tested range of extraction conditions, the total recovery of phenolic compounds varied from 14 to 70 mg GAE g^−1^, whereas the recovery of antioxidants reacting with the DPPH radical ranged from 36 to 319 mg TE g^−1^. Already, at this stage of analysis, it can be assumed that extraction conditions have a significant impact on antioxidant recovery from cranberry leaves (Table 2). However, to accurately assess the effects of individual factors on the total phenolic content (TPC) and antiradical activity (AA), appropriate statistical analyses were performed. Based on the results of a three-factor ANOVA, both linear and quadratic effects were identified as significant contributors to extraction efficiency, with no significant interactions among the tested factors. For polyphenol recovery, a significant quadratic relationship was observed for both ethanol concentration and extraction time. This indicates that increasing the level of a given factor results in a linear increase in response only up to a certain threshold value, beyond which no further increase occurs, or the response may even decrease. In contrast, linear effects were observed for the anti-radical activity of the extracts (Figure 1).

**Figure 2:**
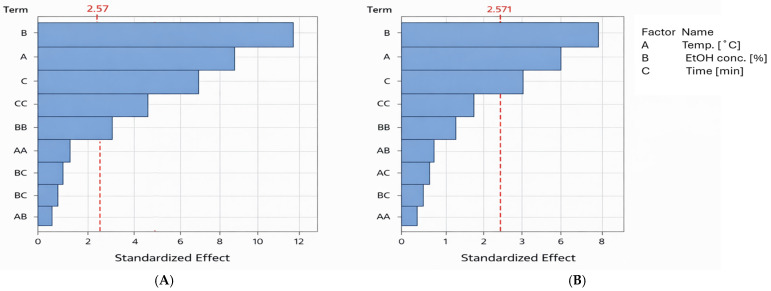
Pareto charts of effects for tested variables. (**A**)—effect of extraction conditions on polyphenol recovery. (**B**)—influence of extraction conditions on antioxidant yield. Abbreviations of terms: A, B, C—the linear effect between analyzed variables; CC, BB, AA—the square effect between analyzed variables; AC, BC, AB—the interaction effect between analyzed variables. The red dotted line indicates the statistical significance threshold. Blue bars (terms) exceeding this line are statistically significant at α = 0.05.

The results of the statistical analysis were also presented as a Pareto chart of effects (Figure 2). This chart illustrates the relative importance of individual factors and interactions on the tested responses (polyphenol recovery and antiradical activity). Bars representing factors that extend beyond the critical vertical line indicate statistically significant effects, and the longer the bar, the greater the influence of the factor on the response. As shown, ethanol concentration has the strongest influence on the recovery of phenol compounds and antioxidants, followed by extraction temperature, and finally extraction time.

Based on these results, the software generated regression model equations describing the observed relationships and enabling the prediction of polyphenol (Equation (1)) and antioxidant (Equation (2)) extraction efficiency within the tested variable range, as well as the determination of optimal process conditions. The equations are as follows:

Y_TPC [mg GAE/g]_ = −9.7 + 1.172A + 0.544B + 0.943C − 0.00506A^2^ − 0.00769B^2^ − 0.00837C^2^ − 0.00093AB − 0.00247AC + 0.00071BC, r^2^ = 98.40%. (1)

Y_AA [mg TE/g]_ = −51 + 3.1A + 2.45B + 5.09C + 0.0188A^2^ − 0.0275B^2^ − 0.0377C^2^ − 0.0173AB − 0.0161AC − 0.0101BC, r^2^ = 96.42% (2)

where A—process temperature [°C], B—ethanol concentration [%, *v*/*v*], and C—process time [min].

The optimal extraction conditions were identified as those providing the highest yield of the analyzed groups of compounds: 40% ethanol, a process temperature of 60 °C, and an extraction time of 49.44 min. To verify these conditions, an additional triplicate extraction was conducted (Table 3). The obtained TPC and AA values did not differ significantly from the calculated values, confirming the accuracy of the regression model. Similar findings were reported by Pérez-Gago et al. [23], who optimized the extraction of wild blueberry leaves and demonstrated that optimal phytochemical yields are achieved using 50% ethanol at 60 °C, as determined through RSM.

Following optimization, the solvent was removed from the liquid extract, which was then subjected to basic phytochemical analysis in its powdered form. The obtained extract was characterized by a high antioxidant activity, ranging from 429 to 490 mg Trolox equivalent per gram, and was composed primarily of phenolic compounds, especially glycoside derivatives of quercetin (Table 4). In the present study, the antioxidant activity values obtained for the pruning biomass extracts are notably high compared to those reported for other cranberry tissues. For instance, Urbstaite et al. reported that ethanolic extracts of *Vaccinium macrocarpon* fruit exhibited radical scavenging activities of up to 849.8 µmol TE g^−1^ and reducing activities of up to 528.1 µmol TE g^−1^ in different cultivars, indicating a substantial antioxidant potential in fruit extracts themselves, albeit at lower magnitudes than observed here in the concentrated pruning biomass [24]. Similarly, studies on cranberry pomace, a by-product of juice processing, have demonstrated a significantly enhanced total polyphenol content and antioxidant capacity compared to whole fruit, with pomace-enriched products showing multiple-fold increases in radical scavenging activity after in vitro digestion [3]. These comparisons highlight the exceptionally high antioxidant potential of extracts obtained from pruning biomass, which may be attributed to the concentration of phenolic compounds and other bioactive constituents in this underutilized plant material, and suggest its strong suitability as a source of natural antioxidants for functional applications.

**Figure 3:**
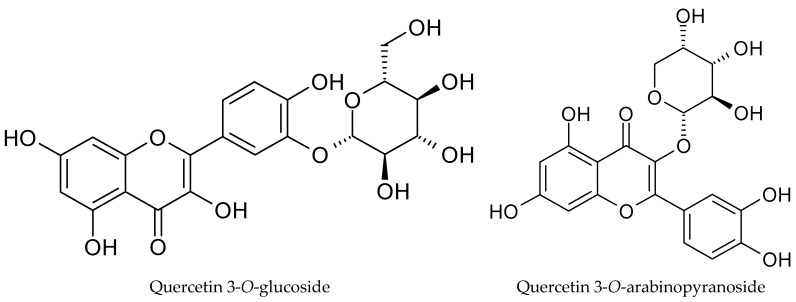
Structures of main detected phenolic compounds.

The phytochemical profile of cranberry leaf extract revealed a clear predominance of quercetin glycosides, with quercetin 3-*O*-glucoside (26.22 ± 0.41 mg TE g^−1^), quercetin 3-*O*-arabinopyranoside (11.73 ± 0.35 mg TE g^−1^), quercetin 3-*O*-rhamnoside (4.16 ± 0.05 mg TE g^−1^), and quercetin 3-*O*-xylopyranoside (2.83 ± 0.05 mg TE g^−1^) being the most abundant phenolic constituents in the extract (Table 4, Figure 3). This distribution is consistent with previous reports indicating that leaves of the *Vaccinium* species are particularly rich in flavonol glycosides, especially quercetin derivatives, which often occur at higher concentrations than in fruit tissues [25]. Quercetin glycosides have attracted considerable interest due to their strong antioxidant and anti-inflammatory activities, which under physiological conditions may surpass those of their aglycone forms. The presence of sugar moieties influences not only the solubility and chemical stability but also intestinal absorption and metabolic fate, thereby determining the overall biological efficacy of these compounds in vivo [26].

The polyphenolic profile obtained in the present study reflects the actual composition of the cranberry leaf raw material used. An untargeted analytical approach was applied, enabling the identification and quantification of all detected compounds above the detection limit using PDA and MS detectors, without restricting the analysis to selected target analytes or reference standards [27]. The corresponding chromatograms obtained using both the PDA detector and the mass spectrometer, as well as the recorded UV–Vis and MS spectra for the identified polyphenolic compounds, are presented in the SM (Supplementary Materials, S1). The identification of nine polyphenolic compounds was carried out using the available standards and literature data as well as the data achieved from MS and MS/MS analyses. The structure of the identified compounds was already determined in some other studies [28,29,30]. Among the anthocyanins, two cyanidin derivatives were identified, all showing the characteristic fragment ion *m*/*z* 287, combined with glucoside and arabinoside moieties. In turn, phenolic acids included 3-*O*-caffeoylquinic acid (chlorogenic) (*m*/*z* 353), which corresponds to coumaroyl dihydromonotropein isomer (*m*/*z* 537). Flavonols were represented by four quercetin derivatives (*m*/*z* 301) and one kaempferol derivative (*m*/*z* 285), characterized by neutral losses corresponding to glucoside (−162 Da), pentose (−132 Da) and rhamnose (−146 Da). Notably, phenolic acids were not detected at significant levels in the extract. This observation may be explained by several factors. Phenolic acids in plant materials typically occur in esterified or glycosylated forms rather than as free acids and are often bound to cell wall polysaccharides, contributing to structural rigidity. Their effective release usually requires acid hydrolysis, which was not applied in the present experimental design. Furthermore, numerous studies have demonstrated that the concentration of phenolic acids decreases during plant growth and maturation. Methodological limitations can be excluded, as the same analytical protocol has been successfully applied to other plant matrices, including berries and dried herbs, where phenolic acids were readily detected. Therefore, the low abundance of phenolic acids observed in this study is most likely attributable to the intrinsic characteristics of the cranberry leaf material rather than analytical constraints.

## 3. Materials and Methods

### 3.1. Plant Material

The plant material used for the study consisted of leaves of the large-fruited cranberry cultivar ‘Pilgrim’, collected in May 2025 from a commercial plantation in Poland (Fieldstone Investments II Sp. z o.o.; Warszawa, Poland) during the plantation periodic rejuvenation.

The cranberry leaves were frozen at −67 °C and then freeze-dried at 28 °C and 150 mTorr for 18 h using a Harvest Right freeze dryer (Salt Lake City, UT, USA). After freeze-drying, the cranberry leaves were ground in a powder mill and stored at −67 °C. The water content of the material was 4.23%, as determined using a moisture analyzer (RADWAG, Radom, Poland).

### 3.2. Extraction Optimization

Extraction optimization was performed using the response surface methodology with a Box–Behnken design. The experimental plan included 15 extractions, in which the variables were: process temperature (25–60 °C), ethanol concentration (40–96%), and extraction time (5–60 min). The experimental design and full statistical analysis were carried out using Minitab software 17 (Minitab Inc., State College, PA, USA).

The extraction process involved weighing 1 g of leaves and adding 40 mL of solvent into an Erlenmeyer flask equipped with a reflux condenser. The flasks containing the extraction mixture were placed in a shaking water bath set to the desired extraction temperature. The extraction was performed in a shaking water bath using horizontal shaking at a frequency of 120 rpm and an amplitude of 25 mm, at a controlled temperature of ±1 °C. To limit oxidative degradation, the extraction vessels were flushed with nitrogen prior to sealing; however, the extraction itself was conducted under atmospheric pressure. Extractions were performed under the conditions specified in Table 1. After the designated extraction time, the extracts were filtered through a 0.45 µm nylon syringe filter and analyzed for total phenolic content and antiradical activity against DPPH^•^.

After conducting ANOVA and regression model (Table 2) analysis—aimed at determining the influence of the factors on antioxidant recovery from cranberry leaves and identifying optimal process conditions—additional triplicate extractions were performed under the established optimal conditions. The extracts were analyzed for total phenolic content and antiradical activity, and the obtained results were compared with the values predicted by the regression model proposed by the software.

**Figure 4:**
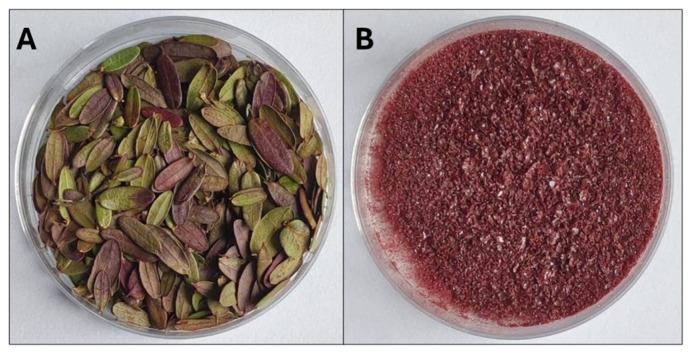
Plant material (cranberry leaves freeze-dried) (**A**) and the extracts obtained from it (**B**).

Finally, the extract obtained under optimal conditions was evaporated to dryness using a rotary evaporator (Heidolph, Schwabach, Germany) (Figure 4B). The residue was weighed to determine the extraction yield. The extract was also subjected to phytochemical analyses, including total phenolic content, antiradical activity, and phenolic compound profiling.

### 3.3. Phytochemical Analysis

The total polyphenol content was measured using the Folin–Ciocalteu reagent in the presence of sodium carbonate based on the protocol presented by Piechowiak and Borowiec [31]. The results were expressed as gallic acid equivalents (GAE). Antiradical activity against DPPH• was analyzed according to the [31] publication, and the results obtained were converted into Trolox equivalents (TE). Both of these parameters were used both to optimize the extraction and to evaluate the phytochemical profile of the solvent-free extract obtained under optimal conditions. The antiradical activity against ABTS^•+^ was assessed using a method that was described in detail in the study by Balawejder et al. [32]. The identification and quantification of individual polyphenolic compounds were performed following the procedure outlined by Zardzewiały et al. [33] using a UPLC–PDA–ESI–MS/MS system (Waters, Milford, MA, USA). Polyphenols were identified and quantified based on their mass-to-charge ratio, retention time, specific PDA spectra, fragmentation patterns (Supplementary Materials) and comparison with both the literature data and commercial standards.

## 4. Conclusions

This study demonstrated that the vegetative cranberry biomass generated during plantation rejuvenation is a valuable yet currently underutilized resource with significant potential for application in technologies aimed at recovering bioactive compounds. The use of response surface methodology (RSM) enabled the optimization of extraction conditions and precise evaluation of the effects of individual process parameters on the efficiency of phenolic compound isolation. The optimal extraction conditions were determined to be 40% ethanol, a temperature of 60 °C, and an extraction time of 49.44 min. The validation of these parameters through an additional triplicate extraction confirmed their effectiveness. The resulting extract was characterized by a high antioxidant activity (429–490 mg Trolox equivalents per gram) and a rich profile of phenolic compounds, with a predominance of quercetin glycoside derivatives. These findings indicate that cranberry pruning biomass may serve as a valuable source of bioactive compounds, and its utilization represents a promising direction for the development of sustainable, environmentally friendly technologies for valorizing agricultural by-products. Further research should be directed towards demonstrating the health-promoting properties of the obtained extracts.

## Figures and Tables

**Table 1:** RSM experimental setup (proposed variants matrix) and results of the analyses obtained.

No	Temperature [°C]	Ethanol Conc. [%, *v*/*v*]	Time [min]	Total Phenolics Level [mg g^−1^] *	Antiradical Activity Against DPPH^•^ [mg g^−1^] **
1	60	40	32.5	70.20 ± 2.79	318.62 ± 29.55
2	42.5	68	32.5	46.27 ± 2.93	187.47 ± 26.91
3	25	40	32.5	45.48 ± 1.16	165.27 ± 7.71
4	25	68	5	22.22 ± 2.63	86.31 ± 5.86
5	42.5	96	5	14.02 ± 0.59	45.14 ± 16.63
6	25	96	32.5	14.91 ± 3.45	36.84 ± 5.48
7	42.5	68	32.5	48.95 ± 4.28	173.58 ± 16.63
8	42.5	40	5	41.68 ± 3.09	147.48 ± 15.17
9	25	68	60	43.27 ± 1.60	164.60 ± 15.51
10	42.5	96	60	34.05 ± 3.25	106.99 ± 3.85
11	42.5	68	32.5	53.81 ± 4.76	194.08 ± 19.38
12	42.5	40	60	59.53 ± 0.43	240.34 ± 12.15
13	60	96	32.5	37.81 ± 2.44	156.29 ± 6.36
14	60	68	60	59.00 ± 4.95	222.71 ± 36.80
15	60	68	5	42.71 ± 3.82	175.44 ± 13.94

*—expressed as gallic acid equivalent (GAE); **—expressed as Trolox equivalent (TE).

**Table 2:** Results of variance analysis (ANOVA) for tested factors and responses.

Source	Total Phenolic Content					Antiradical Activity				
	DF	Adj. SS	Adj. MS	F-Value	*p*-Value	DF	Adj. SS	Adj. MS	F-Value	*p*-Value
**Model**	9	3542.21	393.58	34.22	0.001	9	72,002.10	8000.20	14.95	0.004
Linear	3	3270.80	1090.27	94.79	0.000	3	66,516.90	22,172.32	41.45	0.001
Temperature [°C]	1	878.27	878.72	76.40	0.000	1	22,054.70	22,054.73	41.23	0.001
Ethanol conc. [%]	1	1684.86	1684.86	146.49	0.000	1	34,644.20	34,644.21	64.76	0.000
Time [min]	1	707.22	707.22	61.49	0.001	1	9817.90	9817.91	18.35	0.008
**Square**	3	263.74	87.91	7.64	0.026	3	4717.40	1572.51	2.94	0.138
Temperature^2^ [°C]	1	8.86	8.86	0.77	0.420	1	121.90	121.94	0.23	0.653
Ethanol conc.^2^ [%]	1	134.13	134.13	11.66	0.019	1	1712.00	1712.06	3.20	0.134
Time^2^ [min]	1	148.08	148.08	12.87	0.016	1	3003.80	3003.82	5.62	0.064
**2-way interaction**	3	7.68	2.56	0.22	0.877	3	767.80	255.97	0.48	0.711
Temperature [°C] × Ethanol conc. [%]	1	0.83	0.83	0.07	0.799	1	287.10	287.12	0.54	0.497
Temperature [°C] × Time [min]	1	5.66	5.66	0.49	0.514	1	240.40	240.42	0.45	0.532
Ethanol conc. [%] × Time [min]	1	1.19	1.19	0.10	0.761	1	240.40	240.42	0.45	0.532
**Error**	5	57.51	11.50			3	2674.80	535.07		
Lack of Fit	3	28.33	9.44	0.65	0.654	5	2455.80	818.69	7.47	0.120
Pure Error	2	29.18	14.59			3	219.00	109.57		
**Total**	**14**	**3599.72**				**14**	**74,676.90**			

**Table 3:** Optimal extraction conditions and their impact on the predicted and experimentally determined recovery of total phenols and antioxidants from cranberry leaves. Values marked with the same letter do not differ statistically significantly according to Student’s *t*-test at α = 0.05.

Factors	Temperature [°C]	Ethanol Conc. [%]	Time [min]	Predicted Value		Actual Values	
				TPC [mg GAE g^−1^]	AA [mg TE g^−1^]	TPC [mg GAE g^−1^]	AA [mg TE g^−1^]
**Response**	60	40	49.66	69.86 ^A^	306.79 ^B^	66.94 ± 6.11 ^A^	298.07 ± 10.55 ^B^

**Table 4:** Phytochemical profile in cranberry leaf extract.

Phenolic Compounds Profile (Identified by UPLC-MS)					
Compound	Retention Time (min)	λ_max_ (nm)	[M-H]^+/−^ *m*/*z*		Content (mg g^−1^)
			MS	MS/MS	
**Anthocyanins**					
Cyanidin 3-*O*-glucoside	2.54	279, 512	449^+^	287	1.16 ± 0.02
Cyanidin 3-*O*-arabinoside	2.86	279, 512	419^+^	287	1.02 ± 0.05
**Other phenolics**					
3-*O*-Caffeoylquinic acid	2.43	324	353^−^	191	2.49 ± 0.10
Quercetin 3-*O*-glucoside	5.00	255, 355	463^−^	301	26.22 ± 0.41
Quercetin 3-*O*-xylopyranoside	5.36	255, 355	433^−^	301	2.83 ± 0.05
Coumaroyl-dihydromonotropein	5.49	310	537^−^	163, 119	5.70 ± 0.22
Quercetin 3-*O*-arabinopyranoside	5.69	255, 355	433^−^	301	11.73 ± 0.35
Quercetin 3-*O*-rhamnoside	5.81	255, 352	447^−^	301	4.16 ± 0.05
Kaempferol 3-*O*-glucoside	5.89	267, 352	447^−^	285	1.45 ± 0.07
Total phenolic content (mg GAE g^−1^)					165.98 ± 19.50
Antiradical activity against DPPH^•^ (mg TE g^−1^)					429.95 ± 34.81
Antiradical activity against ABTS^•+^ (mg TE g^−1^)					490.11 ± 26.66
Total extraction yield (g 100 g^−1^)					37.23 ± 4.22

## Data Availability

The original contributions presented in this study are included in the article material. Further inquiries can be directed to the corresponding author.

## Supplementary Materials

The following supporting information can be downloaded at: https://www.mdpi.com/article/10.3390/molecules31040698/s1, Figures S1–S10: Chromatograms and PDA/mass spectra of identified phenolic compounds.
