# Supplementary material for: Unlocking the Potential of Cranberry (Vaccinium macrocarpon Aiton) Pruning Biomass: Phenolic Composition and Antioxidant Properties of Response Surface Methodology Optimized Extracts
Source: Molecules. 2026 Feb 17;31(4):698. doi: 10.3390/molecules31040698 (PMC12943396; doi:10.3390/molecules31040698)
Supplement: Supplementary file 1 [file molecules-31-00698-s001.zip › molecules-4135132-supplementary.pdf]

Supplementary Materials

S1: Chromatograms and PDA/mass spectra of identified phenolic compounds.

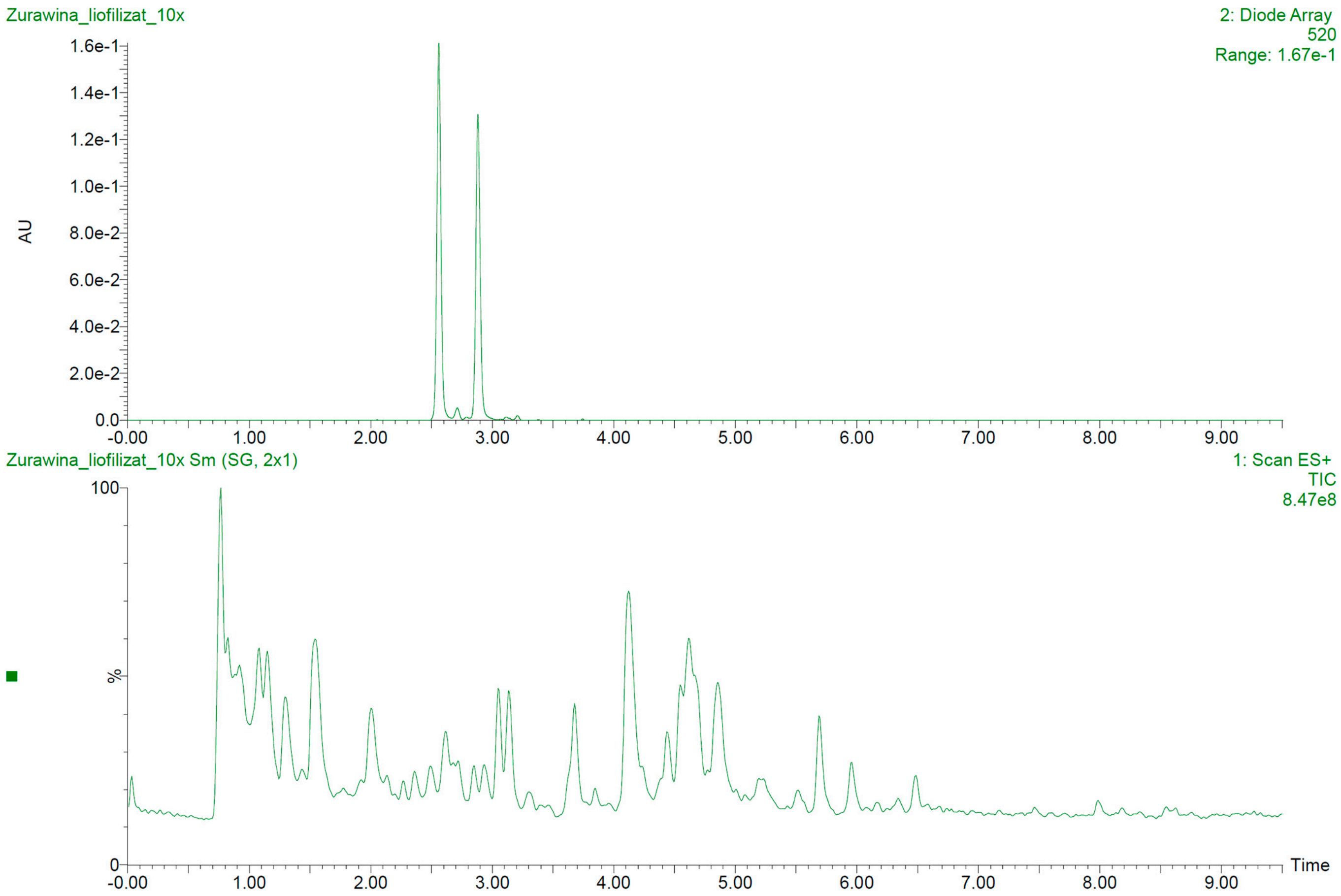

Figure S1. Chromatogram of anthocyanins obtained using PDA (extracted at 520 nm) and TIC.

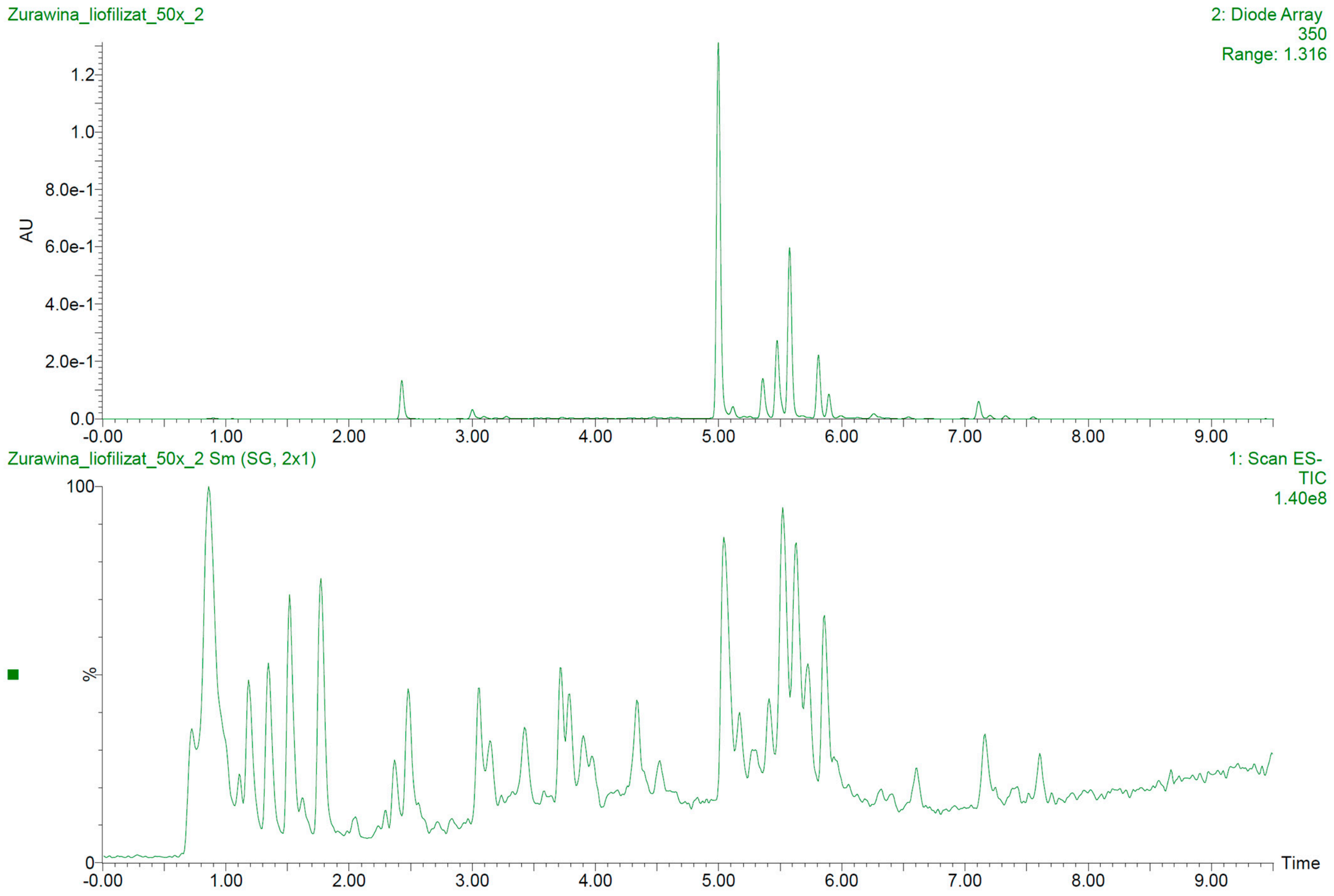

Figure S2. Chromatogram of other phenolics obtained using PDA (extracted at 350 nm) and TIC.

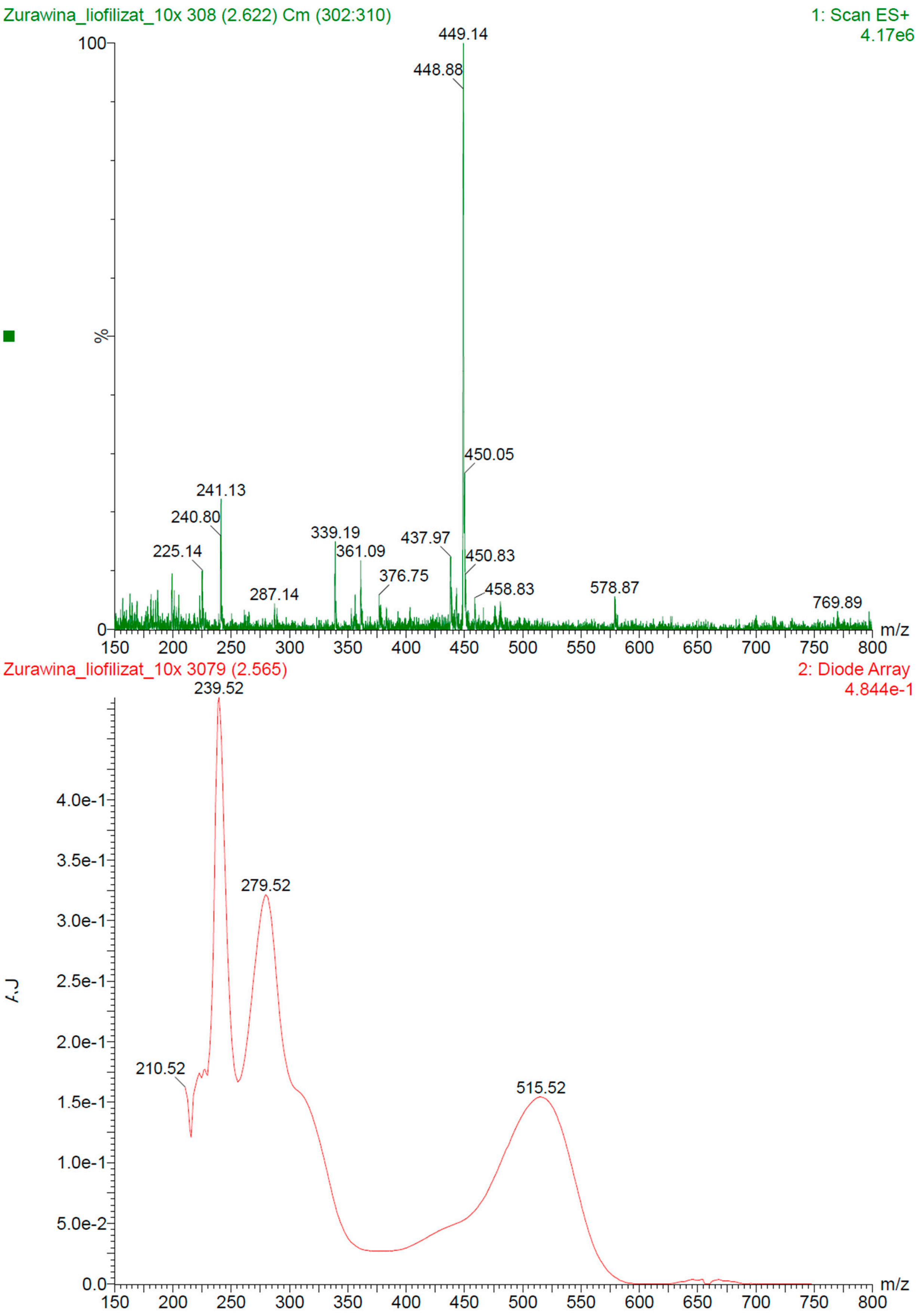

Figure S3. Spectral characterization of cyanidin 3-*O*-glucoside.

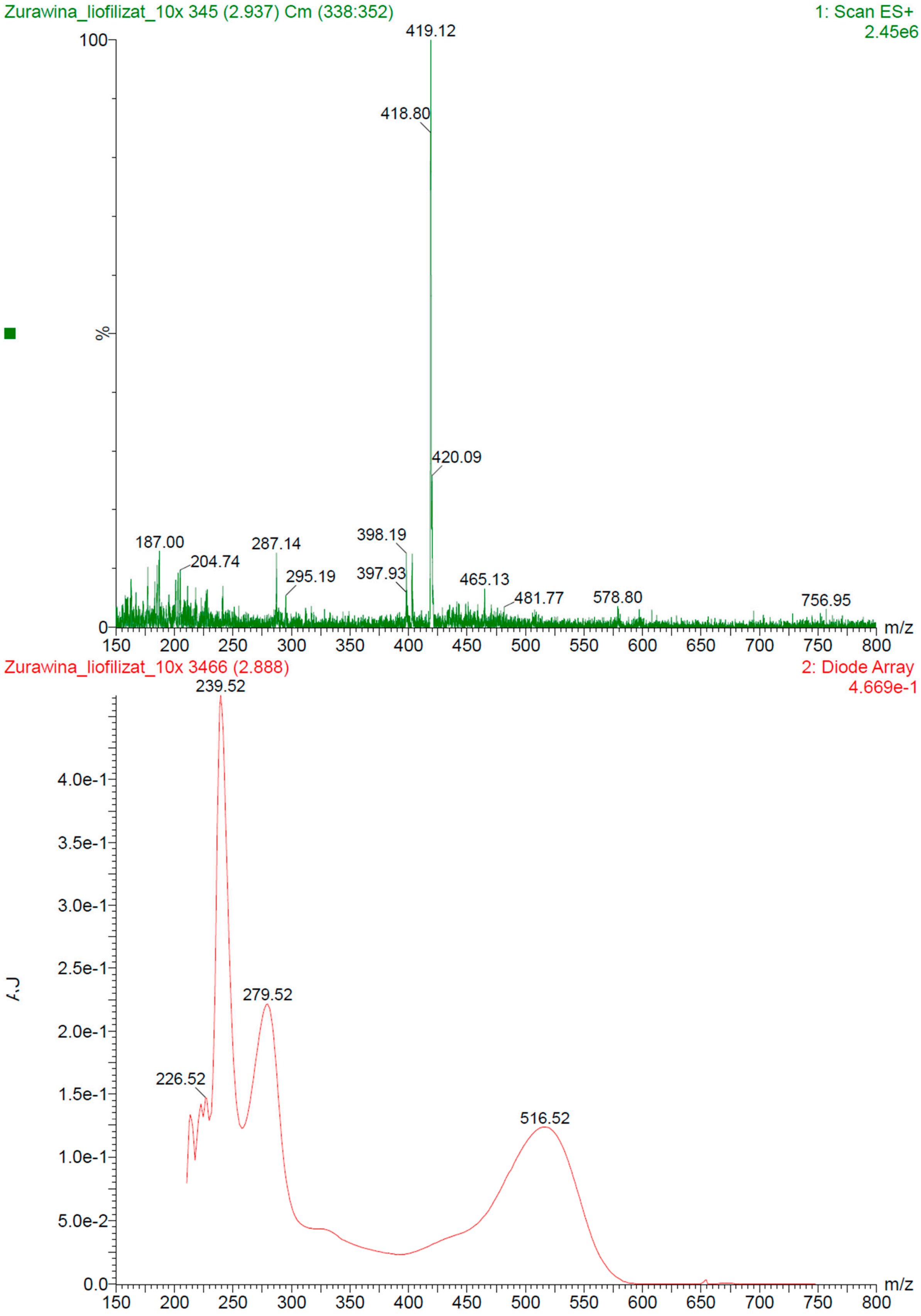

Figure S4. Spectral characterization of cyanidin 3-*O*-arabinoside.

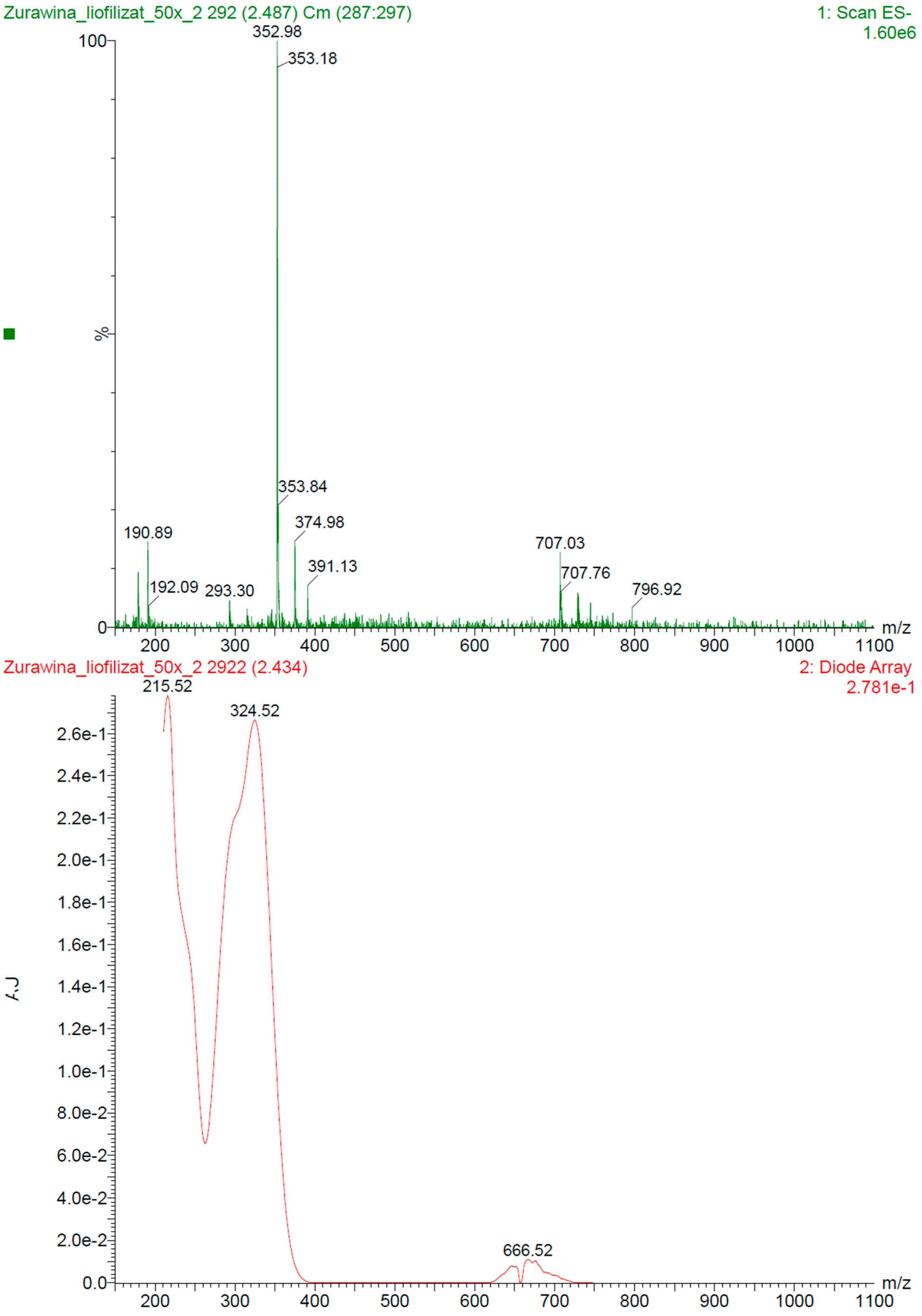

Figure S5. Spectral characterization of 3-*O*-caffeoylquinic acid.

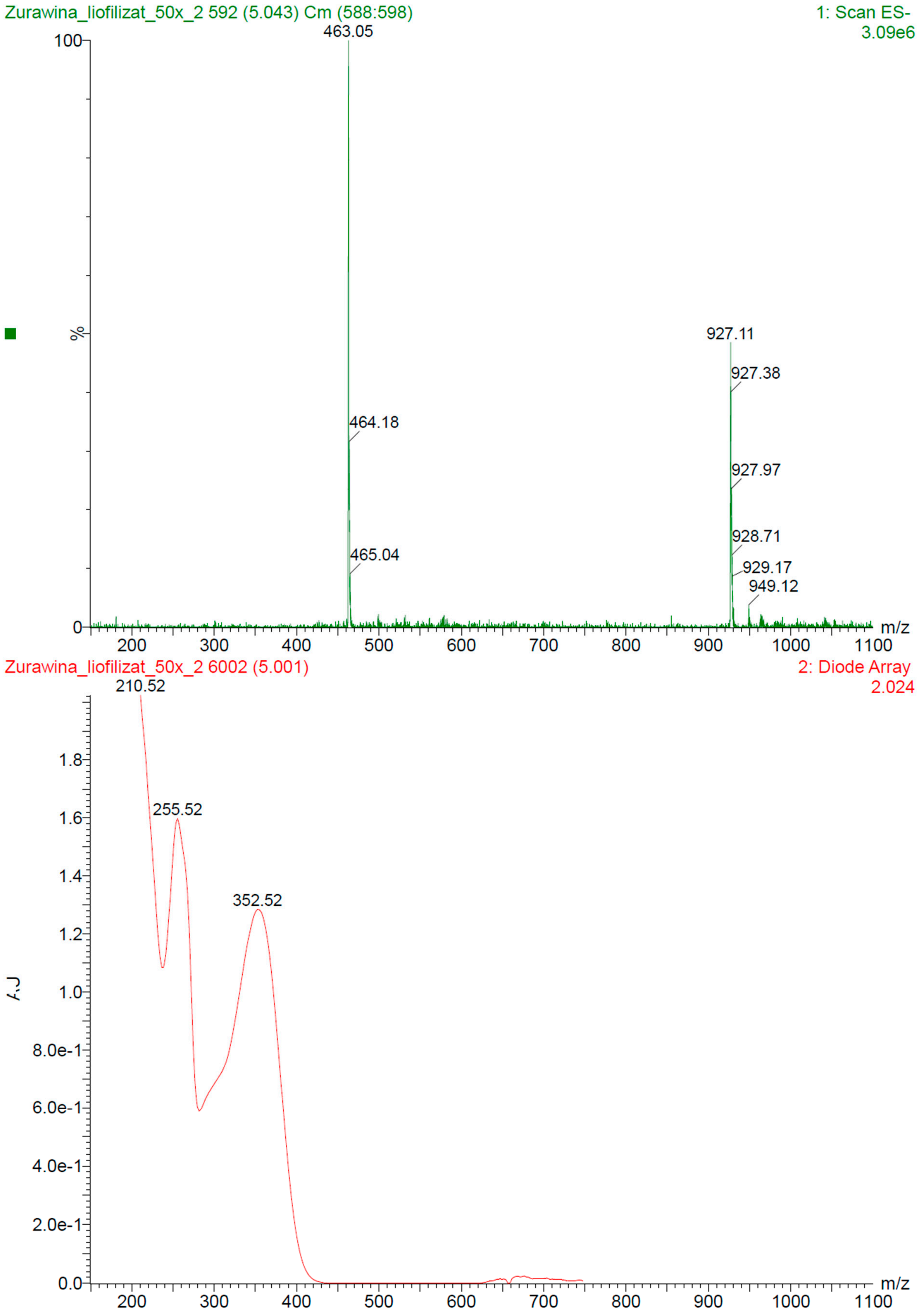

Figure S6. Spectral characterization of quercetin 3-*O*-glucoside.

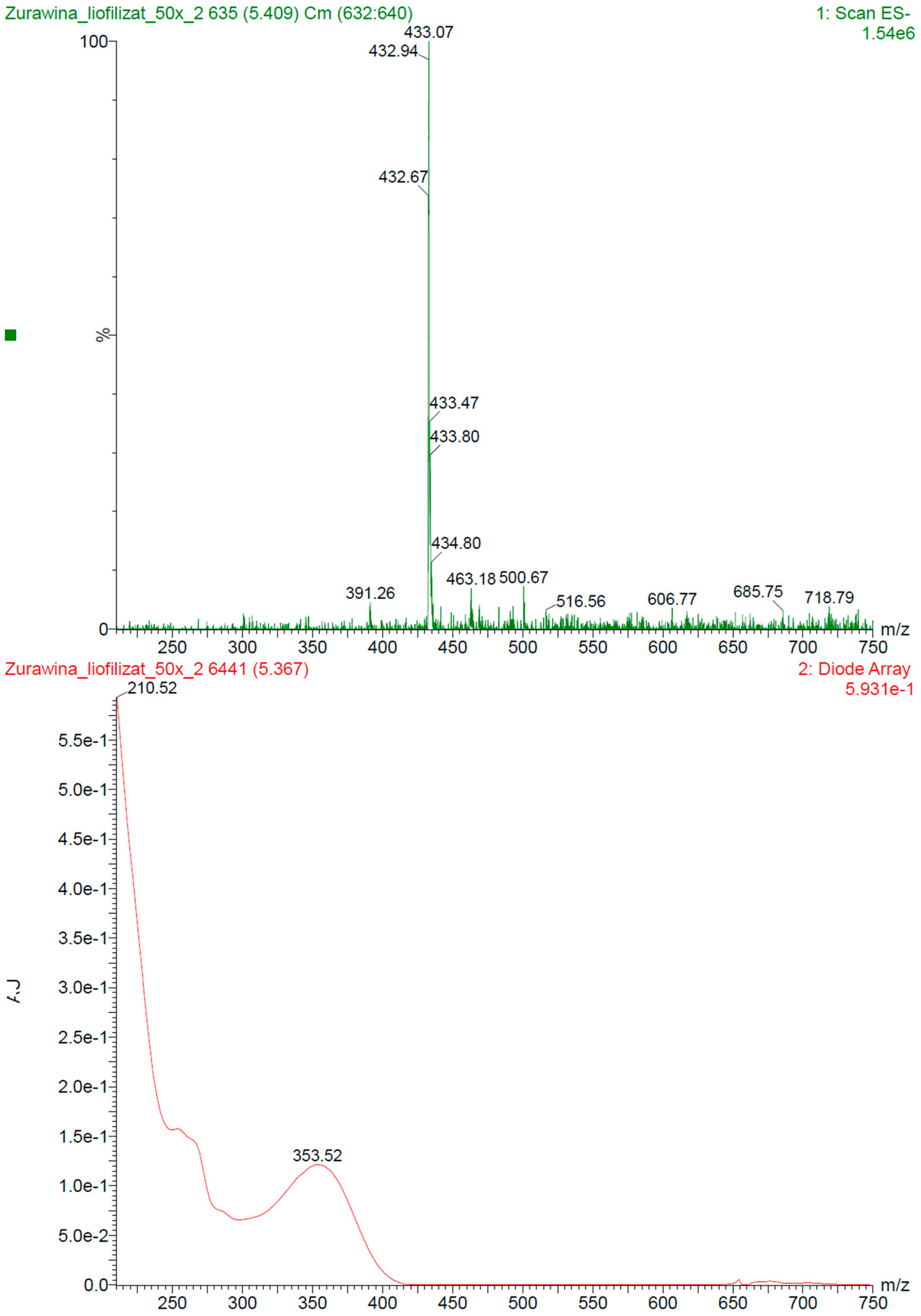

Figure S7. Spectral characterization of quercetin 3-*O*-xylopyranoside and quercetin 3-O-arabinopyranoside.

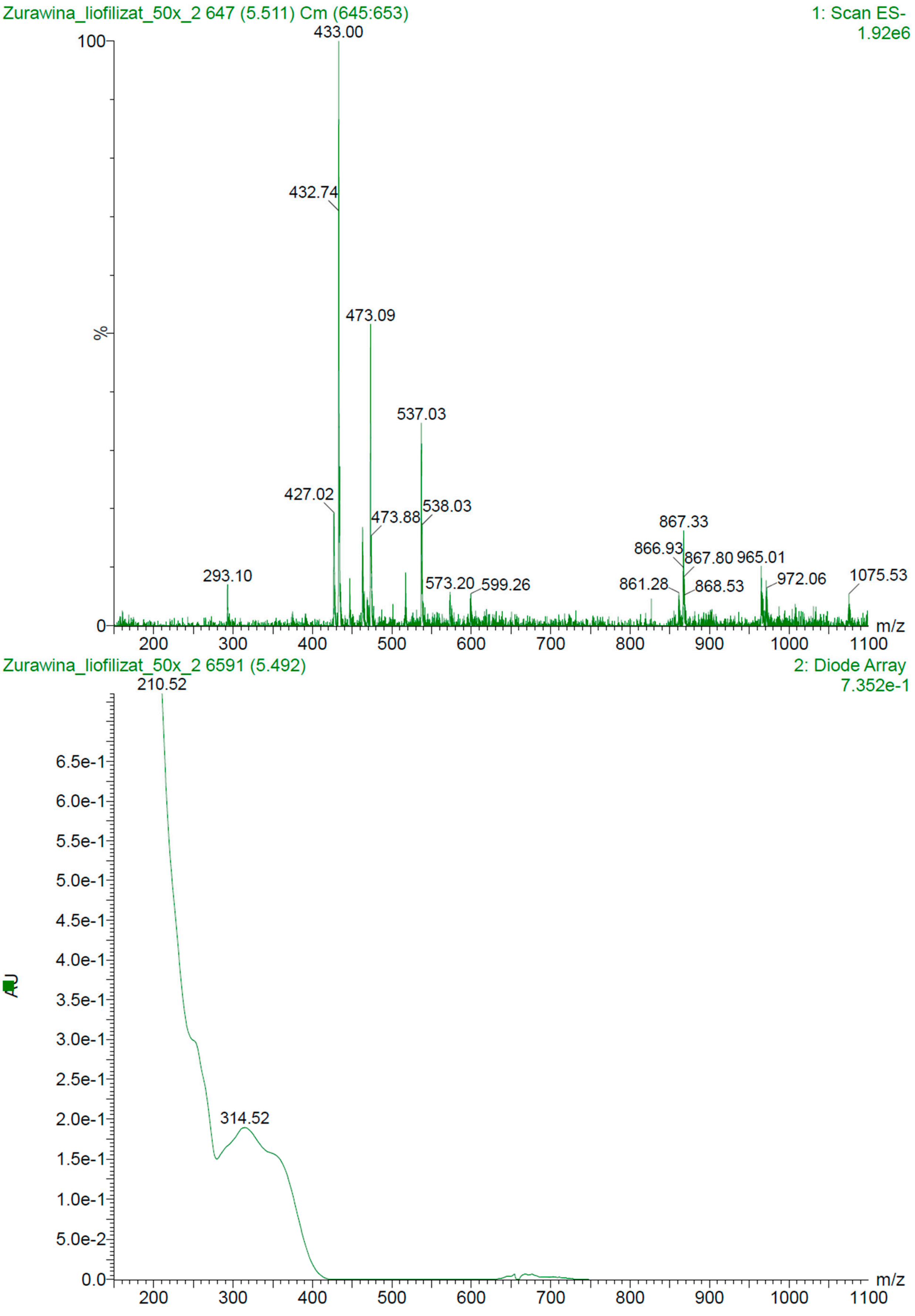

Figure S8. Spectral characterization of Coumaroyl-dihydromonotropein.

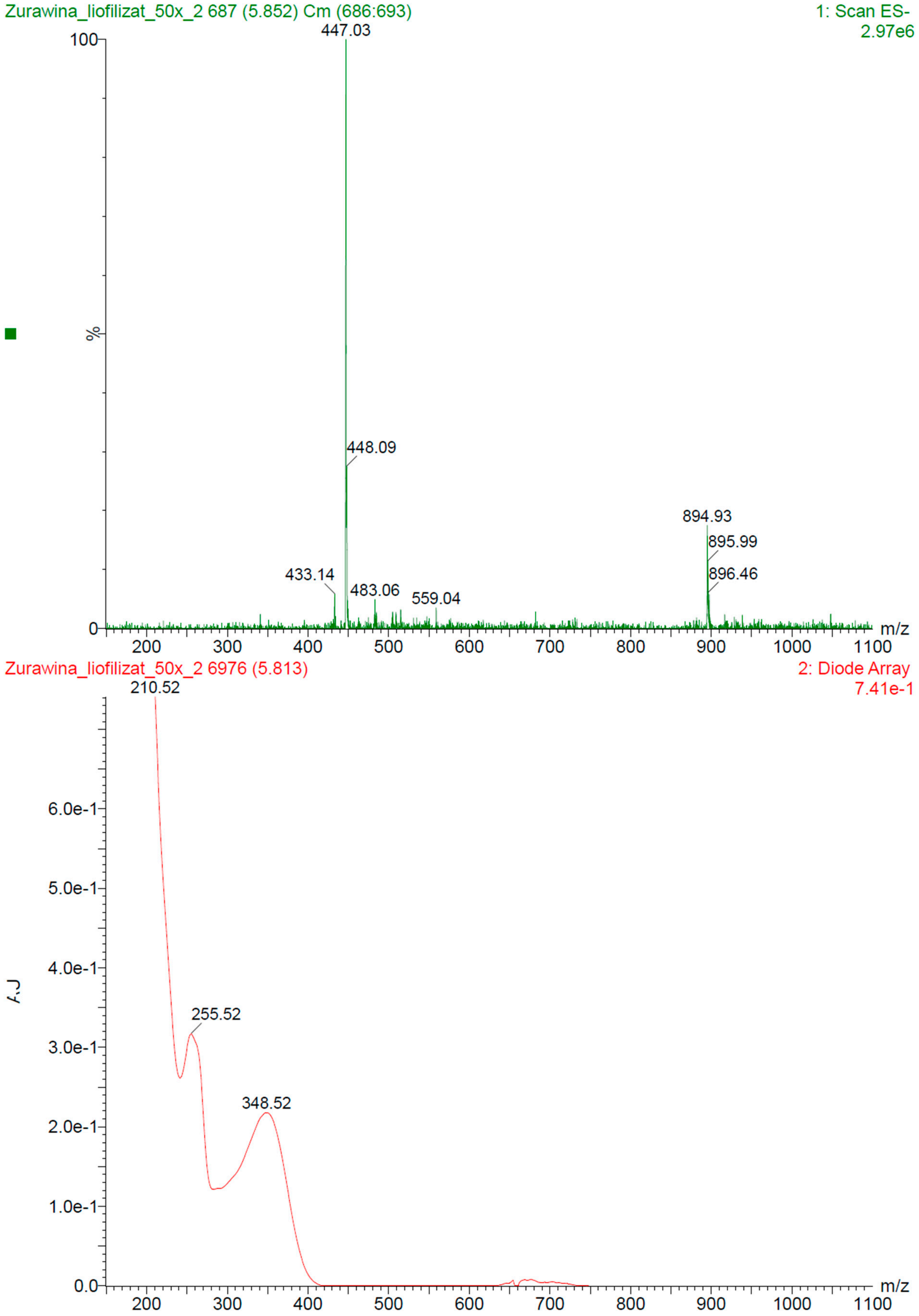

Figure S9. Spectral characterization of quercetin 3-*O*-rhamnoside.

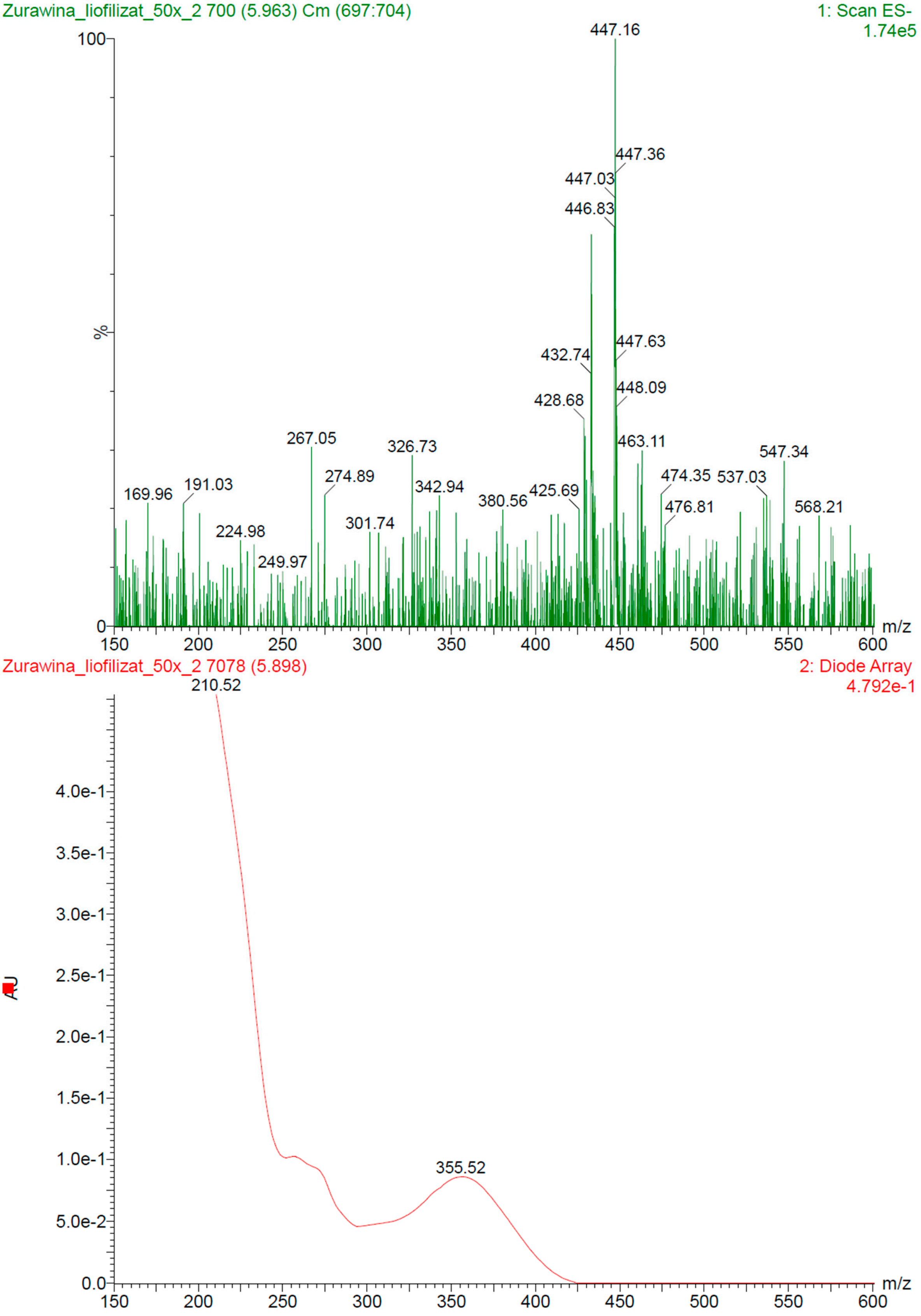

Figure S10. Spectral characterization of kaempferol 3-*O*-glucoside.
